# Imaging in thick samples, a phased Monte Carlo radiation transfer algorithm

**DOI:** 10.1117/1.JBO.26.9.096004

**Published:** 2021-09-07

**Authors:** Lewis McMillan, Sascha Reidt, Cameron McNicol, Isla R. M. Barnard, Michael MacDonald, Christian T. A. Brown, Kenneth Wood

**Affiliations:** a University of St. Andrews, SUPA, School of Physics and Astronomy, St. Andrews, United Kingdom; b University of Dundee, School of Science and Engineering, Dundee, United Kingdom

**Keywords:** Monte Carlo methods, Bessel, scattering, phase, photons, light scattering

## Abstract

**Significance**: Optical microscopy is characterized by the ability to get high resolution, below 1  μm, high contrast, functional and quantitative images. The use of shaped illumination, such as with lightsheet microscopy, has led to greater three-dimensional isotropic resolution with low phototoxicity. However, in most complex samples and tissues, optical imaging is limited by scattering. Many solutions to this issue have been proposed, from using passive approaches such as Bessel beam illumination to active methods incorporating aberration correction, but making fair comparisons between different approaches has proven to be challenging.

**Aim**: We present a phase-encoded Monte Carlo radiation transfer algorithm (φMC) capable of comparing the merits of different illumination strategies or predicting the performance of an individual approach.

**Approach**: We show that φMC is capable of modeling interference phenomena such as Gaussian or Bessel beams and compare the model with experiment.

**Results**: Using this verified model, we show that, for a sample with homogeneously distributed scatterers, there is no inherent advantage to illuminating a sample with a conical wave (Bessel beam) instead of a spherical wave (Gaussian beam), except for maintaining a greater depth of focus.

**Conclusion**: φMC is adaptable to any illumination geometry, sample property, or beam type (such as fractal or layered scatterer distribution) and as such provides a powerful predictive tool for optical imaging in thick samples.

## 1 Introduction

Imaging in thick samples is key to understanding more complex samples, such as developing embryos, organoids, or tissues. Optical imaging is a powerful tool for quantitative and functional imaging but has traditionally struggled with thicker samples where scattering dominates. For example, imaging of zebrafish embryos using lightsheet microscopy is very successful due in large part to the high transparency and low scattering in those particular embryos.^1^^,^^2^ Achieving a greater depth of focus can be achieved using more sophisticated illumination geometries, such as Airy or Bessel beam illumination in lightsheet microscopy.^3^ However, imaging of samples such as chick embyros, which constitutes a more physiologically relevant model for human embryonic development, is greatly hindered by greater levels of scattering in the embryonic tissue.^4^

The standard approach to modeling light propagation in scattering media, from interstellar space to human tissue, is to consider photon transport via a Monte Carlo radiation transfer (MCRT) algorithm.^5^^–^^7^ However, MCRT does not usually preserve the phase state of a photon with each scattering event and as such does not allow for the modeling of coherent scattering or interference effects. Several authors modified the MCRT algorithm in an attempt to model these effects.^8^^–^^11^ However, most of these methods either inaccurately model Gaussian beams, are complex to implement, or carry a heavier computational burden. Most three-dimensional (3D) optical imaging approaches use a shaped illumination field, such as a Gaussian beam, a lightsheet Bessel beam, or an Airy beam. All of these require a spatially coherent illumination source, with the shape of the illumination arising from the interference of, for example, spherical, conical, or cubic phase distributions. We present a phase encoded MCRT (φMC) that preserves the phase of the photons as they scatter in a model tissue, allowing the modeling of arbitrary beam types. This opens up the possibility to make direct and “fair” comparisons between different illumination geometries that are otherwise very challenging to directly compare experimentally.

The use of simple high quality Gaussian beam illumination of a sample, either as a scanning microscopy or in a digitally scanned lightsheet microscopy geometry, has been successful in imaging in a range of different samples.^2^^,^^4^^,^^12^ However, scattering eventually limits the depth to which contrast and resolution can be maintained in an image. Hence, a different approach is needed for these more challenging tissues. Many different approaches to overcoming this challenge have been proposed and demonstrated. These include the direct physical manipulation of the scattering properties via optical clearing of the tissue,^13^^,^^14^ the precompensation of the illumination wavefront to obtain “aberration free” illumination,^15^ and the use of beam types with different wavefronts such as the simple spherical wavefront of a Gaussian beam or more complex shaped light beams such as Airy or Bessel.^3^^,^^16^ Often these approaches are combined, such as the use of deconvolution in tandem with Bessel beam illumination.^17^ What is not always clear is which of these approaches is the most likely to succeed for a sample, or even if these approaches necessarily provide more information on a sample than a simple approach performed well, especially as they inevitably present much greater optical or computational demands.

After first validating on a Young’s slit arrangement and the propagation of Gaussian beam in free space, we apply it to comparing the depth penetration of Bessel and Gaussian beams in homogeneously scattering model tissue. Bessel beams have what are often referred to as “self-healing” properties.^18^ This property arises because the beam, as observed subsequent to an obstruction, is formed by off axis rays that might not have been obstructed themselves. This self-healing is particularly powerful for maintaining optical intensity in the illumination in the presence of not only localized absorption but also localized scattering. However, in a homogeneously scattering medium, in which the complete wavefront of the illuminating beam is affected by the scattering, it is less clear whether or not a conical (Bessel) or spherical (Gaussian) wavefront will be more robust.

Because the energy is distributed so differently in a Bessel beam (between the central maximum and rings) than it is in a diffraction-limited Gaussian beam (all energy in a single spot), it is hard to make direct comparisons between the two. For example, should one compare equal total power in the beam, equal intensity in the Bessel beam’s central spot versus at the focus of the Gaussian beam, or something more physiological and sample specific such as equivalent phototoxicity? This range of approaches makes an experimental comparison very challenging. With such comparisons are made much simpler and subsequently fairer.

## 2 Methods

### 2.1 Monte Carlo Radiation Transfer Method

The radiation transfer equation (RTE) can be used to model the transfer of energy in a medium. However, it generally hard to solve the RTE in arbitrary 3D geometries. The MCRT method can be used to numerically simulate the RTE and calculate various quantities, such as fluence and absorbed energy.

MCRT utilizes random numbers and interaction probabilities to simulate photon transport through media. It is a highly flexible method and can easily model arbitrary 3D geometries, various microphysics including fluorescence and polarization. It can also model various different light sources, from collimated laser beams to diffuse light sources. The only downside to the MCRT method noted in the literature is its computational intensiveness and its lack of ability to model the wave phenomena of light. However, with growing access to high powered computers, this is less of a problem going forward. The second downside, the lack of wave phenomena is what we address in this paper.

Traditionally, MCRT models the particle nature of light via simulating power packets of photons. These simulations allow the modeling of multiple anisotropic scattering and the simulation of various other microphysics. We present our adapted algorithm, φMC, which involved only small modifications from our traditional MCRT algorithm, for modeling light’s interaction with biological tissues,^6^^,^^19^ to simulate the wave behavior of light.

The first of these modifications is the tracking the complex phase of a packet. This is achieved by assigning a phase to the packets on launch, then updating it as the packet moves. The initial phase is described as

$$\varphi =n\;\cos(\frac{2\pi l}{\lambda })+in\;\sin(\frac{2\pi l}{\lambda }),$$ (1)

where φ is the phase, n is the refractive index of the medium, l is the distance the packet has travelled, and λ is the wavelength of a packet.

The second modification to the algorithm is to impose an initial electric field amplitude on each packet. The initial electric field normalizes the power in each packet for N packets. This is achieved via Eq. (2):

$$E_{0}=\frac{1}{N}\sqrt{\frac{P}{A}},$$ (2)

where $E_{0}$ is the initial electric field amplitude, P is the power of the input beam, A is the area of the beam, and N is the number of packets to be simulated. These two modifications allow interference of the different packets to be modeled. We model interference in an area or volume element. We do not model interference at a point where the packets meet; due to the ballistic nature of the packets, this does not occur with enough frequency to give a good signal-to-noise ratio. Equation (3) shows how the interference is modeled for a voxel volume ξ:

$$I(\xi )={\left|\underset{\xi }{\sum }E_{0}\;\cos(\frac{2\pi l}{\lambda })+i\underset{\xi }{\sum }E_{0}\;\sin(\frac{2\pi l}{\lambda })\right|}^{2},\;\xi =(x,y,z),$$ (3)

where I is the intensity, and ξ is the voxel reference, and the other symbols are as before.

The final modification is to use the Huygens–Fresnel principle to generate packet directions. The Huygens–Fresnel principle states that “Every point on a propagating wavefront serves as the source of spherical secondary wavelets…”^20^^–^^22^ The Huygens–Fresnel principle is implemented by sampling the light source on the surface of any lens or in a slit. In practice, this means when, for example, a plane wave is incident on a slit width a and length b, the slit area is uniformly sampled for the initial position of the photon packets. The packets are then given a random direction, sampled toward the detector thus avoiding the nonexistent “backward” waves. For the case of modeling propagation through a lens, the usual geometric optics approach is taken to propagate the packets through the lens. When the packet lies on the surface of the lens, the Huygens–Fresnel principle is invoked, and the packet is given a random direction (in the direction of the medium) and propagated as usual.

These three simple modifications allow the modeling of interference and diffraction in an MCRT simulations.

## 3 Validation

To verify the additions to the MCRT method, we model the double-slit experiment, diffraction through a square aperture in the near and far fields, and show that Gaussian beams and Bessel beams are correctly modeled through free space.

### 3.1 Double-Slit Experiment

**Fig. 1:**
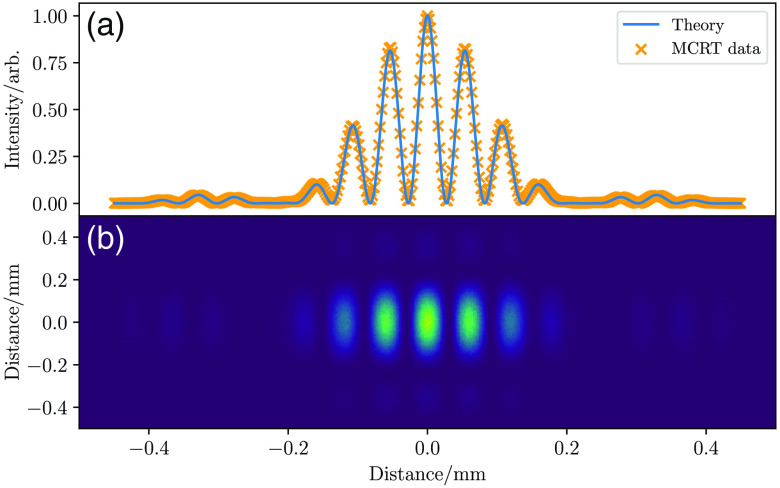
Comparison of theory and simulation for the double-slit experiment. (a) A slice through the computed image and the expected profile from theory. For clarity only every fifth MCRT data point is plotted. (b) The computed image.

The first test of φMC is to compare our algorithm to the double-slit experiment. In the double-slit experiment, a monochromatic plane wave of light is incident on two slits distance apart d, width 2b, and an interference pattern is observed on a screen a distance L away from the slits. The experiment is usually carried out with the detector screen in the far field, the so-called Fraunhofer regime. The intensity pattern on the detector screen is as in Eq. (4):^23^

$$I(x)\propto \cos^{2}(\frac{kdx}{2\sqrt{L^{2}+x^{2}}})\sin c^{2}(\frac{kbx}{\sqrt{L^{2}+x^{2}}}),$$ (4)

where k is the wavevector, $k=\frac{2\pi }{\lambda }$, and x is the horizontal position on the detector screen. The simulation was carried out for a wavelength, λ, of 488 nm, a slit width of 10 λ, slit separation of 80 λ, and the detector screen positioned 10,000 λ away from the slits. Using the Huygens–Fresnel principle, each slit is a source of Huygens wavelets. The detector screen has dimensions, $1\;{\mathrm{mm}}^{2}$ and there are $2051^{2}$ bins, giving a bin an effective size: ≈488  nm or ≈λ. The initial position of the photon packets is sampled uniformly from the slit area, after randomly choosing one of the slits to emit from. A random direction is then chosen to ensure that the packets will hit the detector screen. This is achieved by uniformly sampling a position on the detector screen and calculating the direction vector. The simulation was run with $10^{9}$ packets and the code was parallelized with MPI,^24^ which took ≈10  min to run on an 8 core Intel Xeon machine. This gave an accurate match to the theoretical expression, as shown in Fig. 1.

### 3.2 Diffraction by Square Aperture

We also validate the ability of φMC to simulate wave phenomena in the near field, the so-called Fresnel regime. To test this, we compare results from the simulation against theoretical predictions for diffraction through a square aperture, for both the Fresnel (using the paraxial Fresnel approximation) and Fraunhofer regimes. The Fresnel regime is defined when the Fresnel number, Eq. (5), is greater than 1.0, and the Fraunhofer occurs when the Fresnel number is <1.0. We define the Fresnel number as in Eq. (5),^10^ where l is the slit width, λ is the wavelength, and $r_{0}$ is the distance to the detector screen. As with Young’s slit experiment, the slit is a source of Huygens wavelets. However, we vary the distance to the detector from the square slit such that the Fresnel number changes to validate in the both diffraction regimes:

$$F=l\sqrt{\frac{2}{\lambda r_{0}}}.$$ (5)

**Fig. 2:**
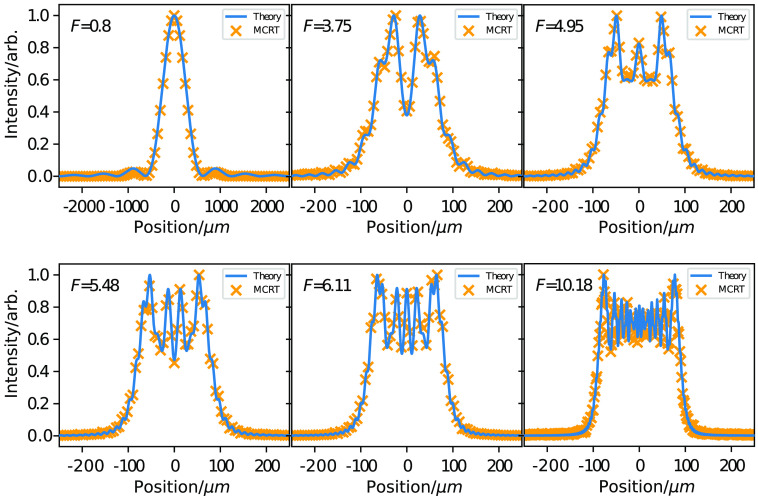
Comparison of theory and simulation for diffraction through a square aperture in the Fresnel and Fraunhofer regimes, for a variety of Fresnel numbers.

We used a wavelength of 351 nm and a slit width/height of 100 nm. For the Fresnel regime, we used 300 bins covering 3600  μm, and for the Fraunhofer, we used 100 bins covering 6000  μm. More bins and photons were required to resolve the complex pattern for higher Fresnel number patterns. Figure 2 shows that matches the theory for both the Fresnel and Fraunhofer regimes.

### 3.3 Gaussian Beams

**Fig. 3:**
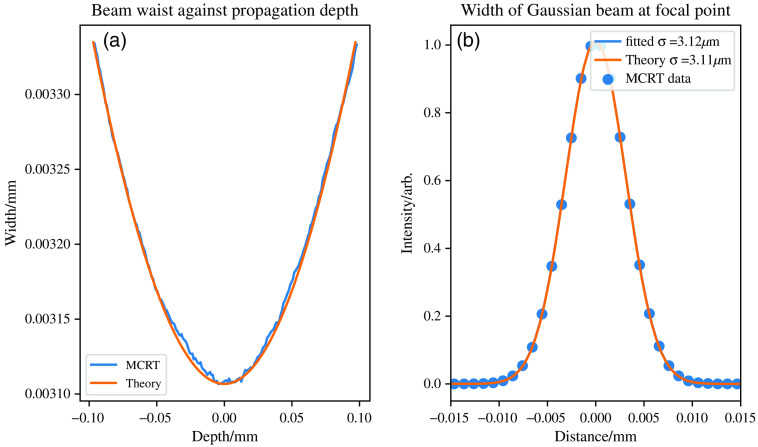
Comparison of Gausssian beam simulation and theory. (a) The Beam waist as a function of distance from its minimum. (b) The width of the Gaussian beam at its minimum.

To show that φMC can model Gaussian beams, we first model a plano-convex lens and focus a Gaussian beam to its focal point and beyond. The plano-convex lens is based upon ThorLabs LA4249 UV-fused silica lens,^25^ which has a radius of 5 mm, thickness of 2.2 mm, and radius of curvature 4.6 mm. A Gaussian beam with $\frac{1}{e^{2}}$ width^23^ 0.5 mm and wavelength 488 nm is incident on the lens. This is propagated through the lens using Snell’s law. When the packet reaches the far surface of the lens, the Huygens–Fresnel principle is used to sample the packet onto the medium’s surface uniformly. The packet is then transported through free space with the usual MCRT method. Figure 3 shows the comparison of theory and *in-silico* experiment, with excellent agreement between the two.

## 4 Bessel Beams

**Fig. 4:**
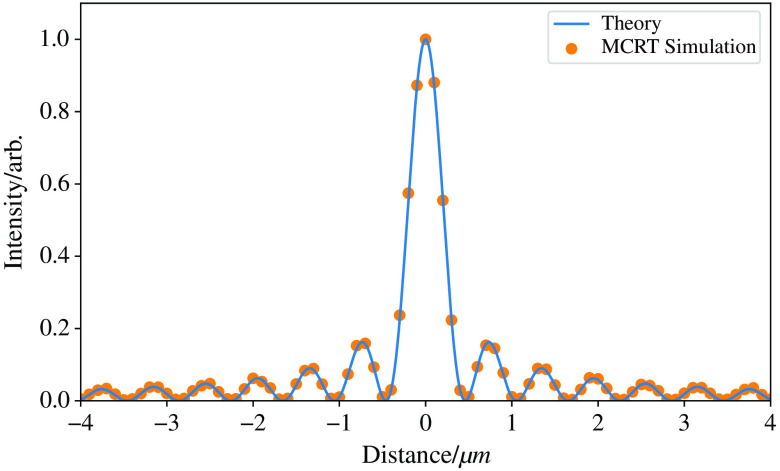
Comparison of theoretical and MCRT simulation of a Bessel beams, with intensity normalized. The results from φMC show good agreement with the theory.

With a little adjustment to the simulation setup, we can also model n’th-order Bessel beams. We change the lens to that of an axicon lens for the 0’th-order beam and to an axicon with a helical delay for a higher order Bessel beams. The axicon used here has an opening angle of 5 deg (α) and radius 12.7 mm. A Gaussian profile beam is incident on the axicon with $1/e^{2}$ width 1 mm. The beam is propagated through free space as described before to a detector screen 10 mm from the tip of the axicon lens. The detector screen has a size of 40  μm×40  μm with a bin resolution of 1  μm. $8\times 10^{10}$ photon packets were simulated taking ∼1  h on an 8-core Intel Xeon 3.5 Ghz machine. Equation (6) gives the equation of a theoretical Bessel beam at a depth zmax normalized. $J_{0}$ is the zeroth-order Bessel function, r is the radial distance, and $k_{r}$ is the radial wavevector. Equation (6) is plotted against the simulation data, with the simulation normalized to the maximum intensity of the image generated. Figure 4 shows this comparison:

$$I(r)=J_{0}^{2}(k_{r},r).$$ (6)

**Fig. 5:**
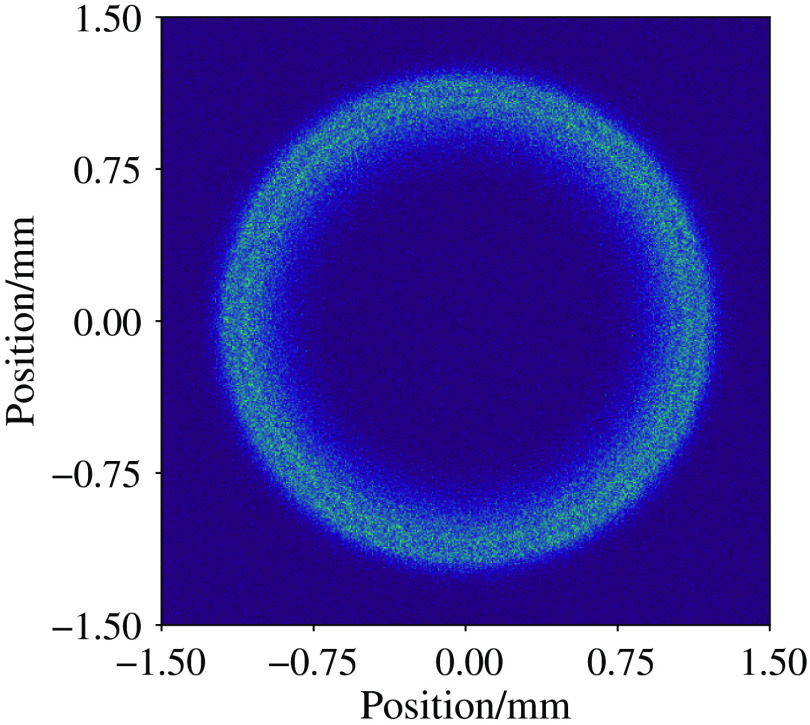
Bessel beam in the far field. Bessel beams in the far field becomes a ring beam. Image shows a slice of intensity through the medium.

Figure 5 shows the profile of the Bessel beam in the far field, where the theory predicts it becomes a ring.

### 4.1 Comparing Simulation to Experiment

**Fig. 6:**
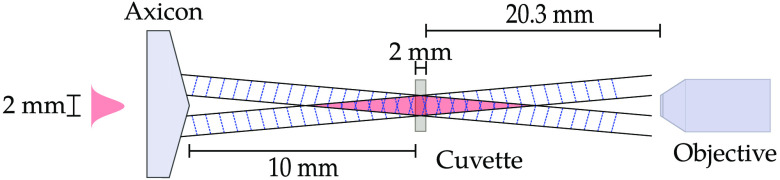
Experimental setup for propagating a Bessel beam through a cuvette filled with varying concentrations of Intralipid 20%. Bessel beam is imaged by an 20× objective lens and a Grasshopper 3 camera.

To ensure our algorithm gets the correct results in turbid media, an experiment was carried in an intralipid scattering agent. The experiments consisted of imaging a Bessel beam after its propagation through an Intralipid medium of varying turbidity. The laser used to create the Bessel beam has a Gaussian profile, and a wavelength of 488 nm, which is incident on an axicon lens with an opening angle 5 deg. The laser beam had a $\frac{1}{e^{2}}$ diameter of 2 mm at the surface of the lens. The Bessel beam was propagated through 10 cm of air before encountering the Intralipid media. The Intralipid was kept in a cuvette of side 2 mm. The cuvette was filled with 500  μL of water, and various volumes of the scattering agent are added. Figure 6 shows the experimental set-up.

**Fig. 7:**
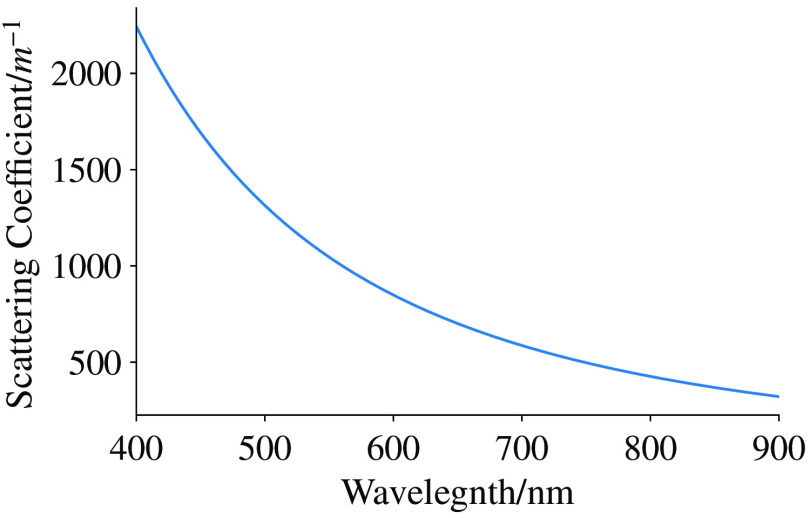
Scattering properties of 20% Intralipid.^26^

The scattering agent used is Intralipid 20% (Sigma-Aldrich), which is diluted as shown in Table 1. Figure 7 shows the optical properties of Intralipid 20%. Dilutions of Intralipid are kept below 2% scattering particle concentration, so the scattering exhibited by the Intralipid is in the independent scattering regime. The independent scattering regime is where g (the anisotropy factor, which is a measure of scattering direction after a scattering event) is dependent on the size, shape, and material properties of the scattering particle, and the material properties of the bulk material but not the number of scattering particles.^27^^,^^28^ This allows the linear scaling of the optical properties by concentration.^27^^,^^29^^,^^30^ Images of the Bessel beam as it emerges from the cuvette are taken for comparison with our algorithm.

**Table 1:** Intralipid solutions used for experiment, see also Fig. 7.

Volume (μL)		Intralipid concentration		Optical properties
Intralipid	$H_{2}O$	Volume (%)	Scattering particle (%)	Scattering coefficient ($m^{-1}$)
0	500	0.00	0.00	0.00
2	500	0.39841	0.0908	557.14
4	500	0.79365	0.1816	1114.28
6	500	1.18577	0.2724	1671.42
8	500	1.57480	0.3632	2228.56

To model within φMC, we simplify the experimental setup considerably. The simulation models the propagation of photon packets through the axicon to its conical surface. On the conical surface, the Huygens–Fresnel principle is invoked, and the packet is sampled onto the surface of the medium (cuvette). The sampling of the photon onto the surface of the medium speeds the algorithm up, as it does not need to simulate the photons that would “miss” the medium. From there, the usual MCRT method propagates the packet through the medium while tracking its phase, and scattering the packet until it leaves the medium. If the packet leaves the medium to any side other than the far side of the cuvette (e.g., any side of the cuvette not facing the objective lens), then it is discarded. If the packet leaves the medium on the objective lens facing side, then the packet is recorded by its phase onto an area element. For each Intralipid concentration $6.4\times 10^{10}$ photons are run over 64 cores, taking ∼3  h for the 12-μL Intralipid volume. Once all the packets have been run, the phase is converted into intensity, as in Eq. (3), but in two-dimensional.

**Fig. 8:**
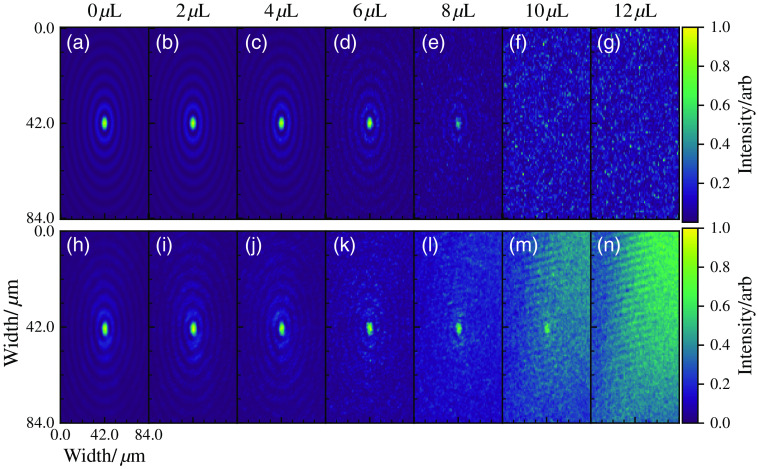
Comparison of experimental and simulation data for propagation of a Bessel beam produced by an axicon, through mediums of various turbidity. (a)–(g) The data from φMC, and (h)–(n) the experimental data. Volumes along the top are the volume of Intralipid in each solution as in Table 1. All images are cropped so they are the same size and normalized to the maximum value in each image.

**Fig. 9:**
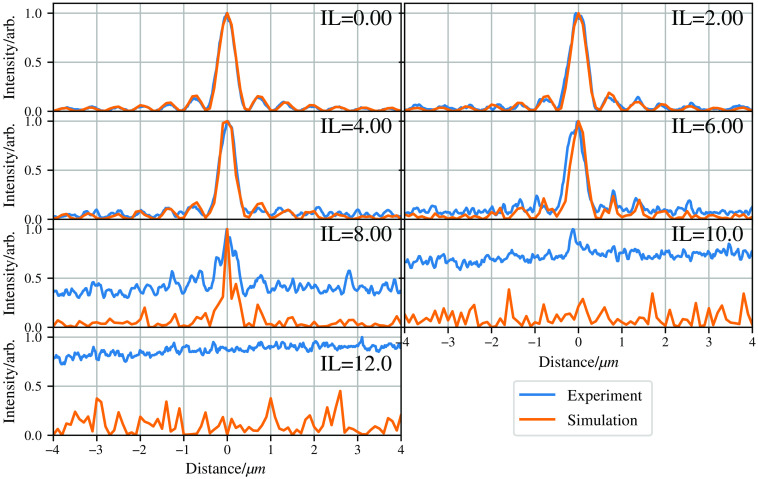
Line graph plots of slices taken through the generated and experimental images as shown in Fig. 8.

Figures 8 and 9 show the results from the experiment and simulation. To allow for the comparison between the experimental and *in-silico* data, we normalized each image to its brightest pixel. The simulations show good agreement with the experimental data within experimental and simulation uncertainty.

## 5 Comparison of Bessel and Gaussian Beams

As Bessel and Gaussian beams are radically different from one another, it is hard to directly compare the two beams. Gaussian beams carry all their power in the “central core” of the beam, whereas in a Bessel beam, it carries the same amount of power in each ring. Bessel beams also have a much larger depth of focus than Gaussian beams. We attempt to give a fair comparison of the two beams, to predict which beam performs better in a heavily scattering medium using φMC. Bessel beams are expected to perform better than Gaussian beams, due to their self-healing properties and nondiffractive core, this section aims to quantify how this property may or may not help penetration through a highly scattering medium.

As mentioned, Bessel beams and Gaussian beams are not alike, so to ensure a fair comparison the Bessel beams central core width [Eq. (7)] is set to that of the Gaussian beam’s waist:

$$r_{0}=\frac{\kappa }{k\;\sin\;\beta },$$ (7)

where κ is a constant that determines the metric used to measure the Bessel beam’s core, $r_{0}$ is the Bessel beams central cores width, k is the wavenumber, and β is the angle that a ray of is deflected by the axicon. For κ=2.408, the radius is measured from the maximum of the core to the first zero of the Bessel function. κ=1.75 measures the Bessel beam’s core from the maximum to $\frac{1}{e^{2}}$ of the maximum, we therefore use this metric to compare the core size with that of the Gaussian beams width. For both beams central cores to be equal in width, the axicon used to generate the Bessel beam is adjusted. This is achieved by calculating the “correct” α based upon the optical setup used to focus the Gaussian beam. Using the small angle approximation, for small α and β: β=(n−1)α, and κ=1.75 we can compare the Bessel beam’s core radius to a Gaussian beam’s waist:^23^^,^^31^

$$\frac{1.75\lambda }{2\pi \;\sin\;\beta }=\frac{2\lambda f}{\pi D},$$ (8)

$$\alpha =\frac{1}{n-1}a\;\sin(\frac{1.75D}{4f}),$$ (9)

where α is the axicon angle as before, n is the refractive index of the axicon, D is the $\frac{1}{e^{2}}$ diameter of the incident Gaussian beam on the lens, and f is the focal length of the lens used to focus the Gaussian beam. Both D and f are the properties of the optical system used to focus the Gaussian beam. The lens used to focus the Gaussian beam is the same as used in the previous section to model a Gaussian beam in φMC, a convex-plano lens, with radius of curvature 4.6 mm, a working distance of 8.5 mm, and thickness of 2.2 mm.

**Fig. 10:**
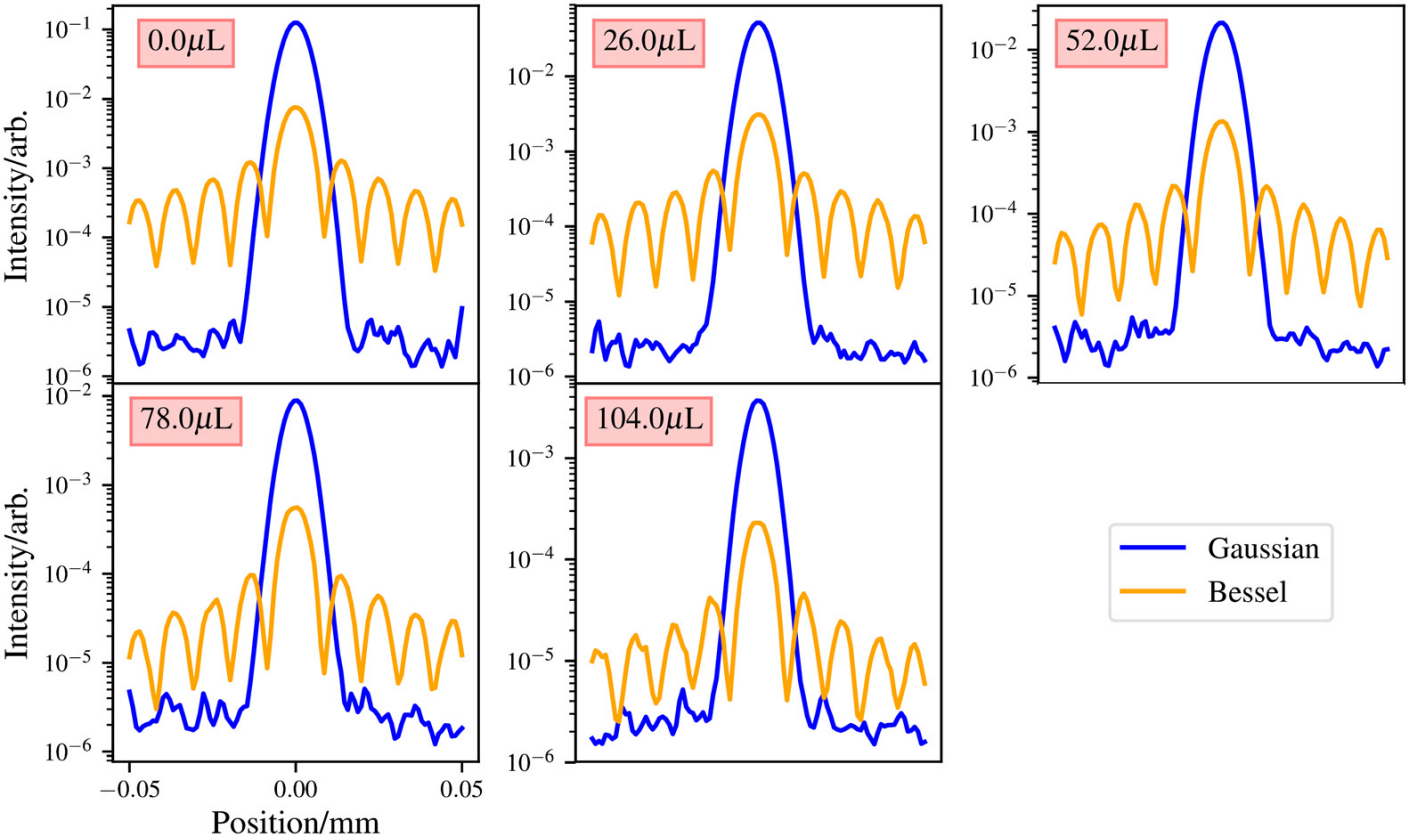
First comparison of Bessel and Gaussian beams with equal power used to generate both beams. Plots taken at the Gaussian beams focus. The maxima at the sides of the Gaussian beam in the 0.0  μL plot are due to simulation effects, mainly the small size of the medium not allowing photons from further off the optical axis to interfere destructively.

**Fig. 11:**
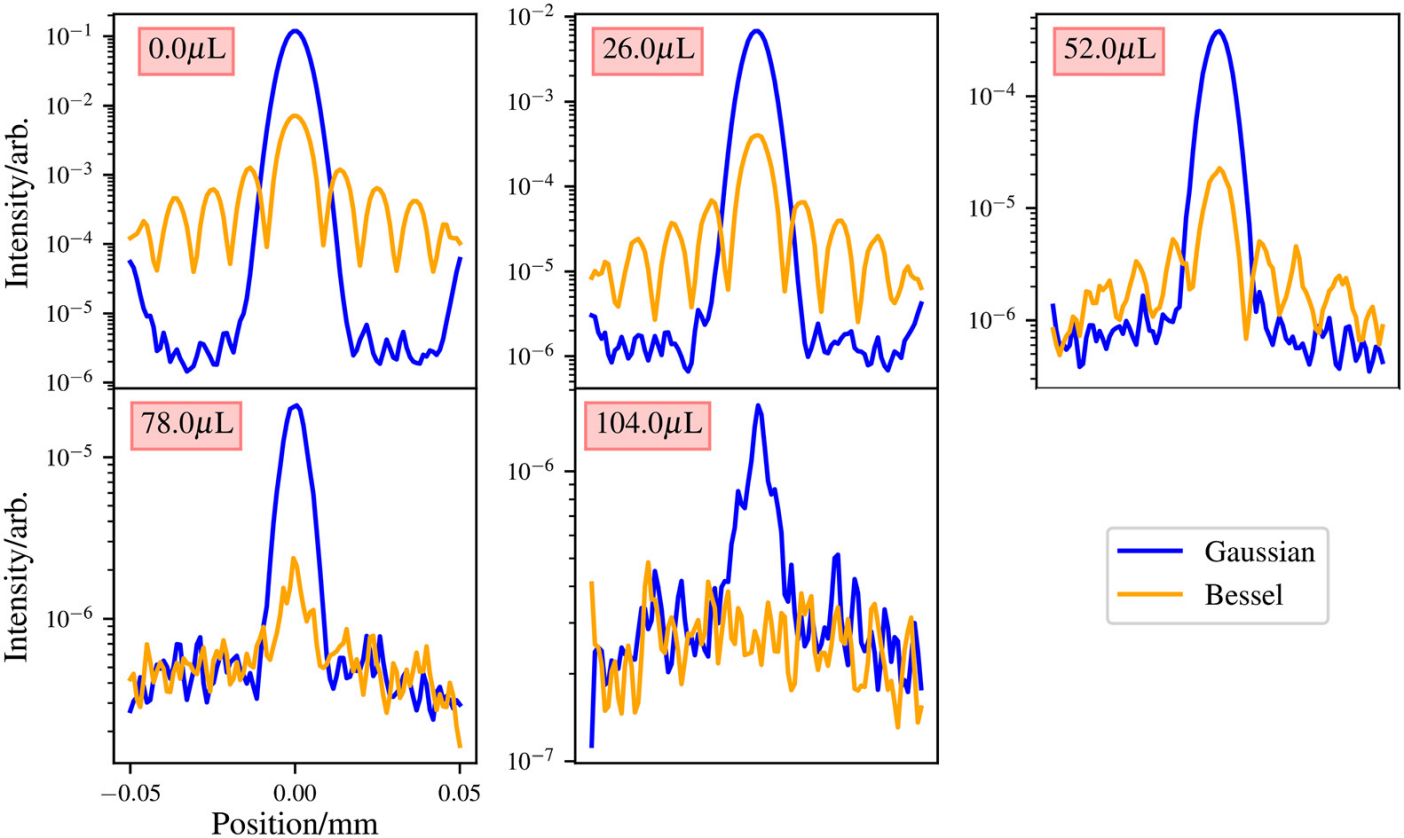
First comparison of Bessel and Gaussian beams, with equal power used to generate both beams. Plots taken at the bottom of the simulated medium. Medium has a 2 mm thickness.

The first simulation comparisons carried out between the Bessel and Gaussian beams is to use the same power to generate both beams. The beams are then propagated through mediums of varying degrees of Intralipid solution. Volumes of 0.0, 26, 52, 78, and 104  μL are used of Intralipid in 500  μL of water. The medium has a volume of 0.1  mm×0.1  mm×0.2  mm, and voxel resolution of 1  μm. For both beams, a wavelength of 488 nm and a power of 1 mW is used. One hundred million packets are simulated for each simulation. The results of this are shown in Figs. 10 and 11.

**Fig. 12:**
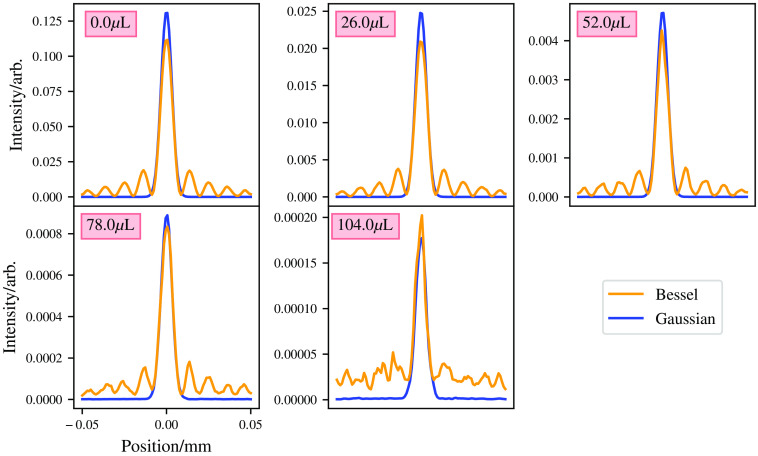
Second comparison of Bessel and Gaussian beams for the case where the power given to each beam yields the same maximum at the Gaussian beams focus. These plots are taken from the Gaussian beams focus. Medium has a 2-mm thickness.

The results show that for the same power, Gaussian beams propagate deeper into the medium compared with Bessel beams. This is to be expected as in a Gaussian beam all the power is in its “central core,” whereas the power is evenly distributed between all the Bessel beam’s rings. Therefore, for a second comparison, the power given to the Bessel beam is such that the central core maximum matches that of the Gaussian beam at its focus for the case where there is no scattering. To achieve this, the Bessel beam was given ∼15× the power given to the Gaussian beam. The results of this comparison are illustrated in Fig. 12.

These results show as expected that the Bessel beam now performs comparably with the Gaussian beam in lower scattering media, with a drop off in performance in the higher scattering media.

## 6 Discussion

For equal power beams in the previous section, Gaussian beams perform “better” in the highly scattering media, though this is expected as the power in a Bessel beam is spread evenly over its rings. Thus, the power in the central lobe of a Bessel beam is much less than that of the Gaussian beam.

**Fig. 13:**
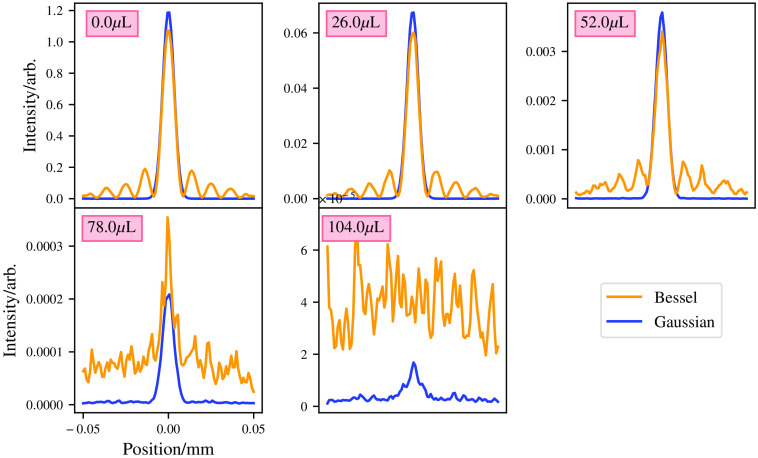
Comparisons of unequal powered beams at the bottom of scattering medium. Medium has a 2-mm thickness.

To give a slightly fairer comparison of intensity in the central lobes of the beams, the Bessel beam was given 15× more power than the Gaussian beam. This allows a better comparison between the Gaussian beam’s core and the Bessel beam’s core and gives a more comparable intensity between the beam’s at the location of the Gaussian beams focus. In this case, the Bessel beam appears to perform better in a highly scattering medium, as shown in Fig. 13. The Bessel beam shows comparable intensity with the Gaussian beam in the first three mediums, though the Gaussian beam out performs the Bessel beam in the higher scattering media. It would appear that the Bessel beams self-healing property does not help a Bessel beam propagate through a highly scattering medium. As photons propagate through the medium, they interfere with one another constructively and destructively to form a Bessel beam. However, if enough photons are scattered, then the Bessel beam becomes degraded and thus no longer is a Bessel beam, as these photons are no longer coherent with the rest of the beam, so they act as a negative factor in the beams formation. Another reason that the “self-healing” property of the Bessel beam does not “save” the beam from scattering is that the “self-healing” is not self-healing. The self-healing in reality is just photons from further off the optical axis forming the Bessel beam further down the optical axis, e.g., the photons that are impeded by the blockage are stopped, but the photons that are not impeded form a Bessel beam as expected. If you placed a blockage in front of the Bessel beam larger than the width of the input beam, the Bessel beam would not form at all.

Bessel beams do have their positives, their self-healing property does help “reform” the beam past small blockages, and their depth of field is superior to an equivalent Gaussian beam, as their central core is “nondiffractive.”

This technique is fairly computationally heavy due to the stochastic nature of the simulations. However, with the advent of fast graphical processing unit, this computational cost going forward is not the barrier it may have once posed.

## 7 Conclusion

This work presents an adaption to the Monte Carlo radiative transfer algorithm to allow the simulation of the wave properties of light. We showed that MCRT can be easily modified to allow the simulation of interference of light. These additions to the MCRT algorithm allowed a fair comparison between Gaussian and Bessel beams, something that is not easily achievable in a lab setting. We showed that despite Bessel beams self-healing property, we found that it does not outperform a comparable Gaussian beam when it comes to imaging at depth. However, the Bessel beams’ depth of focus is still vastly better than that of the Gaussian beam.

## Data, Materials, and Code Availability

Code available at: http://doi.org/10.5281/zenodo.4782307. Data available at: http://doi.org/10.5281/zenodo.4782292.
